# HucMSC exosome-delivered 14-3-3ζ alleviates ultraviolet radiation-induced photodamage via SIRT1 pathway modulation

**DOI:** 10.18632/aging.202851

**Published:** 2021-04-21

**Authors:** Peipei Wu, Bin Zhang, Xinye Han, Yaoxiang Sun, Zixuan Sun, Linli Li, Xinru Zhou, Qian Jin, Peiwen Fu, Wenrong Xu, Hui Qian

**Affiliations:** 1 Key Laboratory of Laboratory Medicine of Jiangsu Province, School of Medicine, Jiangsu University, Zhenjiang 212013, Jiangsu, People’s Republic of China; 2 Zhenjiang Key Laboratory of High Technology Research on Exosomes Foundation and Transformation Application, Jiangsu Key Laboratory of Medical Science and Laboratory Medicine, School of Medicine, Jiangsu University, Zhenjiang 212013, Jiangsu, People’s Republic of China; 3 Department of Laboratory Medicine, Affiliated Hospital of Jining Medical University, Jining 272000, Shandong, People’s Republic of China

**Keywords:** exosomes, SIRT1, mesenchymal stem cells, DNA damage, oxidative stress

## Abstract

Exosomes derived from human umbilical cord mesenchymal stem cells (hucMSC-ex) are nano-sized membrane-bound vesicles that have been reported to facilitate skin regeneration and repair. However, the roles played by hucMSC-ex in ultraviolet (UV) radiation-induced skin photodamage and the underlying mechanisms remain unknown. To investigate the functions of hucMSC-ex in a rat model of acute skin photodamage, immunofluorescence and immunohistochemical staining, quantitative real-time-polymerase chain reaction (qRT-PCR), western blot, and gene silencing assays were performed. We found that the *in vivo* subcutaneous injection of hucMSC-ex elicited antioxidant and anti-inflammatory effects against UV radiation-induced DNA damage and apoptosis. Further studies showed that the sirtuin 1 (SIRT1) expression level in skin keratinocytes (HaCaT) decreased in a time- and dose-dependent manner under *in vitro* UV radiation induced-oxidative stress conditions, which could be reversed by treatment with hucMSC-ex. The activation of SIRT1 significantly attenuated UV- and H_2_O_2_-induced cytotoxic damage by inhibiting oxidative stress and promoting the activation of autophagy. Our study found that 14-3-3ζ protein, which was delivered by hucMSC-ex, exerted a cytoprotective function via the modulation of a SIRT1-dependent antioxidant pathway. Collectively, our findings indicated that hucMSC-ex might represent a new potential agent for preventing or treating UV radiation-induced skin photodamage and aging.

## INTRODUCTION

Ultraviolet (UV) radiation is one of the most important environmental risk factors for skin damage. The increased deterioration of the ozone layer has significantly increased exposure of human skin to solar UV radiation. Exposure to UV radiation can stimulate the generation of large amounts of reactive oxygen species (ROS), which can induce lipid peroxidation, protein modifications, and DNA damage. UV radiation can also promote apoptosis-related signal transduction. Long-term exposure to excessive UV radiation can result in epidermal and dermal stem cell depletion and damage the stem cell microenvironment. Together, these factors eventually trigger a series of pathological processes, including photodamage, photoaging, and the development of photo-induced malignant skin tumors [1–3].

Emerging evidence has shown that transplanted mesenchymal stem cells (MSCs) may represent a promising strategy for cutaneous regeneration and repair [4]. The therapeutic effects associated with MSC transplantation *in vivo* are mediated primarily by paracrine signaling [5–8]. However, MSC transplantation can be complicated by ethical issues and risks of tumorigenic mutations [9]. Exosomes are small membrane-bound vesicles that originate from the intralumenal budding of the late endosomal membrane and deliver various functional proteins and nucleic acid materials (DNA, mRNA, and microRNA [miRNA]) to the recipient cells to modulate their activity [10, 11]. In recent years, numerous studies have confirmed that exosomes are key paracrine components in MSCs, producing effects similar to those exerted by parental MSCs. Our previous studies demonstrated that exosomes derived from human umbilical cord mesenchymal stem cells (hucMSC-ex) have positive therapeutic effects under various disease conditions, including acute myocardial ischemia–reperfusion injury [12], liver fibrosis [13], and cutaneous wound healing [14–16]. However, whether hucMSC-ex can protect against UV radiation-induced acute skin photodamage remains unclear.

Silent information regulator 1 (SIRT1), the mammalian orthologue of SIR2, an NAD-dependent class III histone deacetylase [17], plays a crucial role in multiple biological processes, including inflammation, cell metabolism, cellular senescence, oxidative stress, cell death, and survival [18]. Whether the hucMSC-ex mediated activation of the SIRT1 pathway can alleviate UV radiation-induced skin damage is currently unknown. In this study, we evaluated the feasibility and efficacy of hucMSC-ex treatment for the alleviation of UV radiation induced in a skin photodamage model. Our study showed that hucMSC-ex treatment could reduce redness, scaling, and inflammatory cell infiltration in the skin. We also found that hucMSC-ex treatment promoted the detoxification of H_2_O_2_, repressed DNA damage, and inhibited apoptosis *in vitro* and *in vivo*. The cytoprotective effects exerted by the hucMSC-ex-derived 14-3-3ζ protein might be associated with the modulation of the SIRT1-dependent antioxidant response. Our results provide a better understanding of the use of hucMSC-ex to treat skin photodamage.

## RESULTS

### The characteristics of hucMSCs and hucMSC-ex

**Figure 1:**
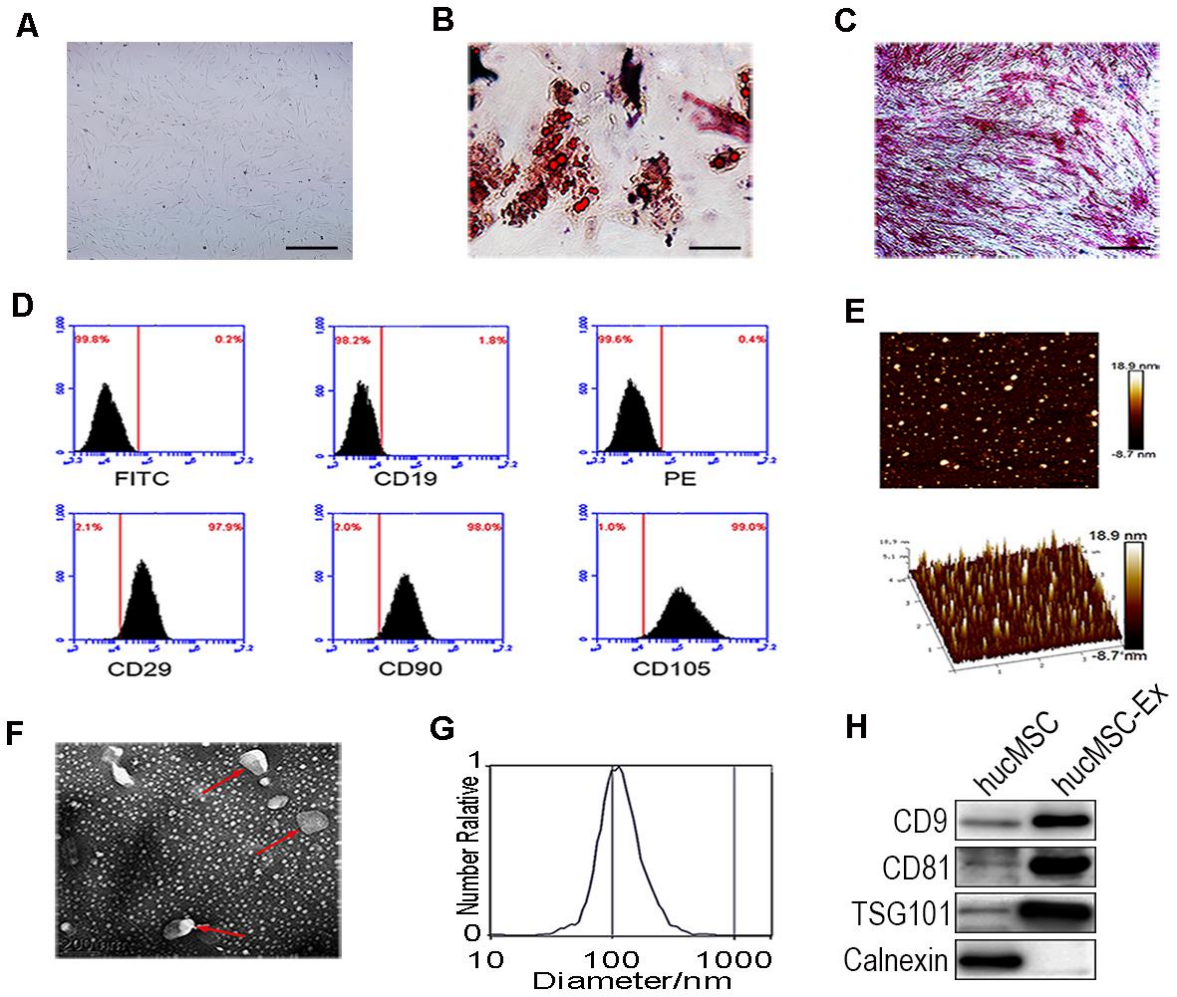
**Identification of hucMSC and hucMSC-ex.** (**A**) Morphological identification of hucMSC. (**B**) Adipogenic differentiation of hucMSC. Adipogenic differentiation was analyzed by Oil-Red-O staining. (**C**) Osteogenic differentiation of hucMSC was shown by neutrophil alkaline phosphatase (NAP) staining (100x). (**D**) Flow cytometry analyses of phenotypic markers of hucMSC:CD19, CD29, CD90, CD105. (**E**) Representative AFM image of hucMSC-ex. (**F**) Representative TEM image of hucMSC-ex (Scale bar=200 nm). (**G**) The hucMSC-ex size distribution were analyzed using nanoparticle tracking analysis (NTA) with ZetaView_Particle Metrix. (**H**) Detection of hucMSC-ex surface marker expression by western blot.

HucMSCs were isolated and purified, as previously described (Figure 1A). After induction in osteogenic and adipogenic medium, Oil-Red-O staining revealed numerous lipid droplets in the HucMSC cytoplasm (Figure 1B), and the cells became alkaline phosphatase positive (Figure 1C). These results showed that hucMSCs could be differentiated into both adipocytes and osteoblasts. Fluorescence-activated cell sorting (FACS) analysis demonstrated that hucMSCs highly express typical MSC surface markers, including CD29 (97.9%), CD90 (98.0%), and CD105 (99.0%), with the low expression of the B lymphocyte surface marker CD19 (1.8%, Figure 1D). HucMSC-ex were isolated and purified from the cell culture supernatant, as previously described. Atomic force microscope (AFM), Transmission electron microscope (TEM), nanoparticle tracking analysis (NTA) and western blot assays were used to identify the morphology, particle size distributions and counts, and surface marker expression levels of the isolated exosomes, respectively. AFM, TEM and NTA revealed that hucMSC-ex displayed a classical spherical morphology (Figure 1E, 1F), approximately 40–100 nm in diameter (Figure 1G). The western blot results indicated that hucMSC-ex expressed exosome-specific biomarkers, such as CD9, CD81, tumor susceptibility gene 101 (TSG101) but did not express calnexin (Figure 1H).

### HucMSC-ex ameliorates UV radiation-induced skin photodamage

**Figure 2:**
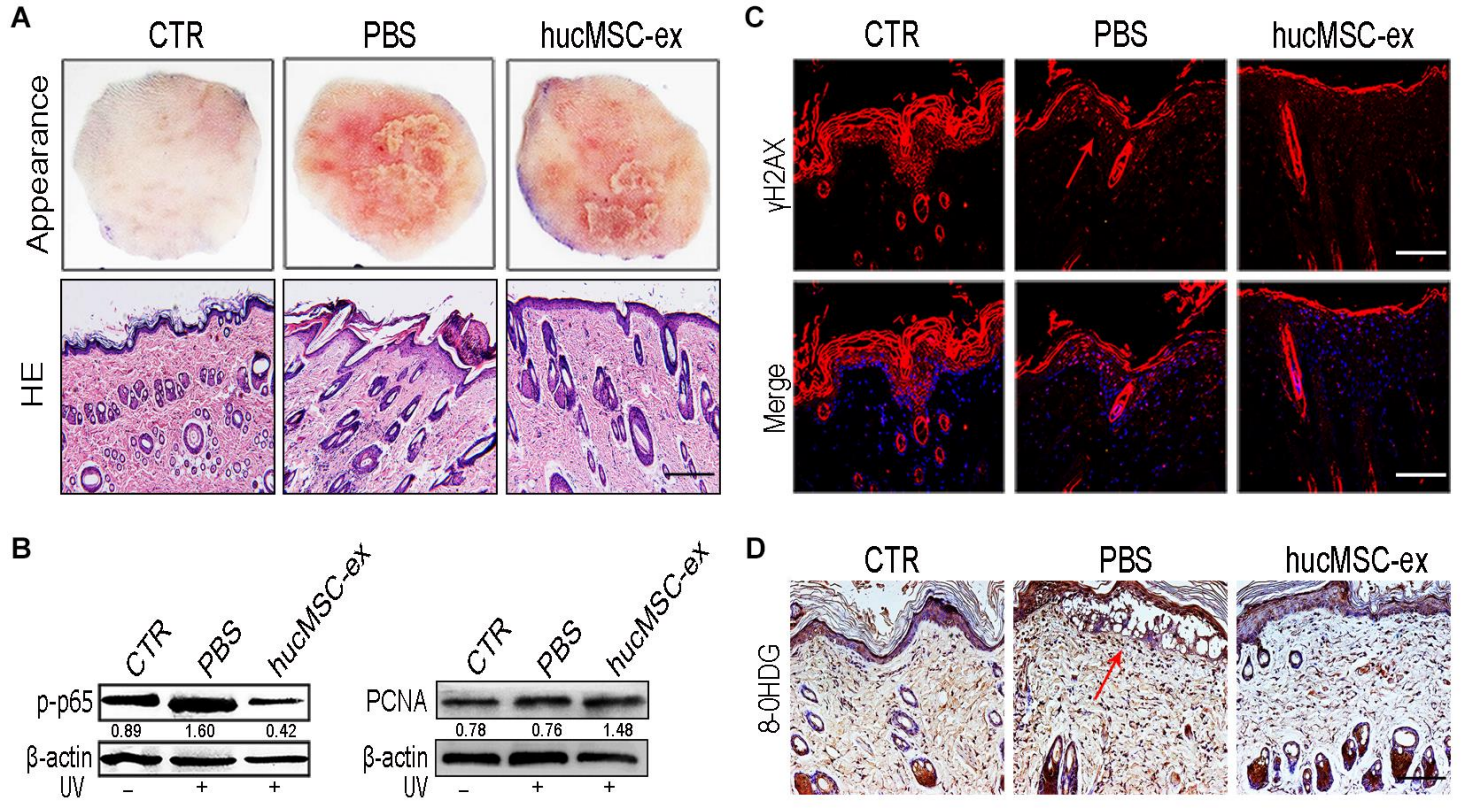
**HucMSC-ex protect skin cell from oxidative stress *in vivo*.** (**A**) Representative images of skin appearance and H&E staining after hucMSC-ex treatment 72 hours significantly inhibited skin inflammation and accelerated skin cell renewal (n=5). Original magnification (100x). (**B**) p-NF-κB and PCNA expression level quantified by western blot. (**C**, **D**): Immunofluorescence and immunohistochemical staining analysis of cutaneous tissues γH2AX and 8-OHDG expression level, Original magnification (200x).

To investigate whether hucMSC-ex treatment could exert any protective effects against UV radiation-induced skin photodamage, we established a rat model of acute photodamage. The histologic appearance of the skin and hematoxylin and eosin (H&E) staining showed that hucMSC-ex treatment with subcutaneous injection significantly reduced skin inflammation and promoted skin cell regeneration (Figure 2A). We detected the relative expression levels of p-p65 and proliferating cell nuclear antigen (PCNA) using western blot analysis and found that hucMSC-ex treatment significantly decreased the level of p-p65 expression and increased the level of PCNA expression in the rat model of UV radiation-induced acute skin damage (Figure 2B).

UV irradiation is one of the most harmful factors to which skin can be exposed, inducing ROS, oxidative stress, and cytotoxicity, causing sunburn, erythema, photoaging, and photocarcinogenesis. To illustrate the antioxidant effects of hucMSC-ex, we examined the phosphorylated histone 2Ax (γH2AX) and 8-hydroxy-2′-deoxyguanosine (8-OHDG), which are produced in response to the oxidative damage of DNA. The immunohistochemical staining results showed that in the phosphate-buffered saline (PBS)-treated group, the DNA damage markers γH2AX and 8-OHDG were notably increased. However, their levels decreased significantly in the hucMSC-ex-treated group (Figure 2C, 2D). These results demonstrated that the ability of the hucMSC-ex treatment to relieve UV radiation-induced skin damage might be associated with antioxidant components contained in hucMSC-ex.

### HucMSC-ex significantly reduces UV radiation-induced ROS production *in vitro*

**Figure 3:**
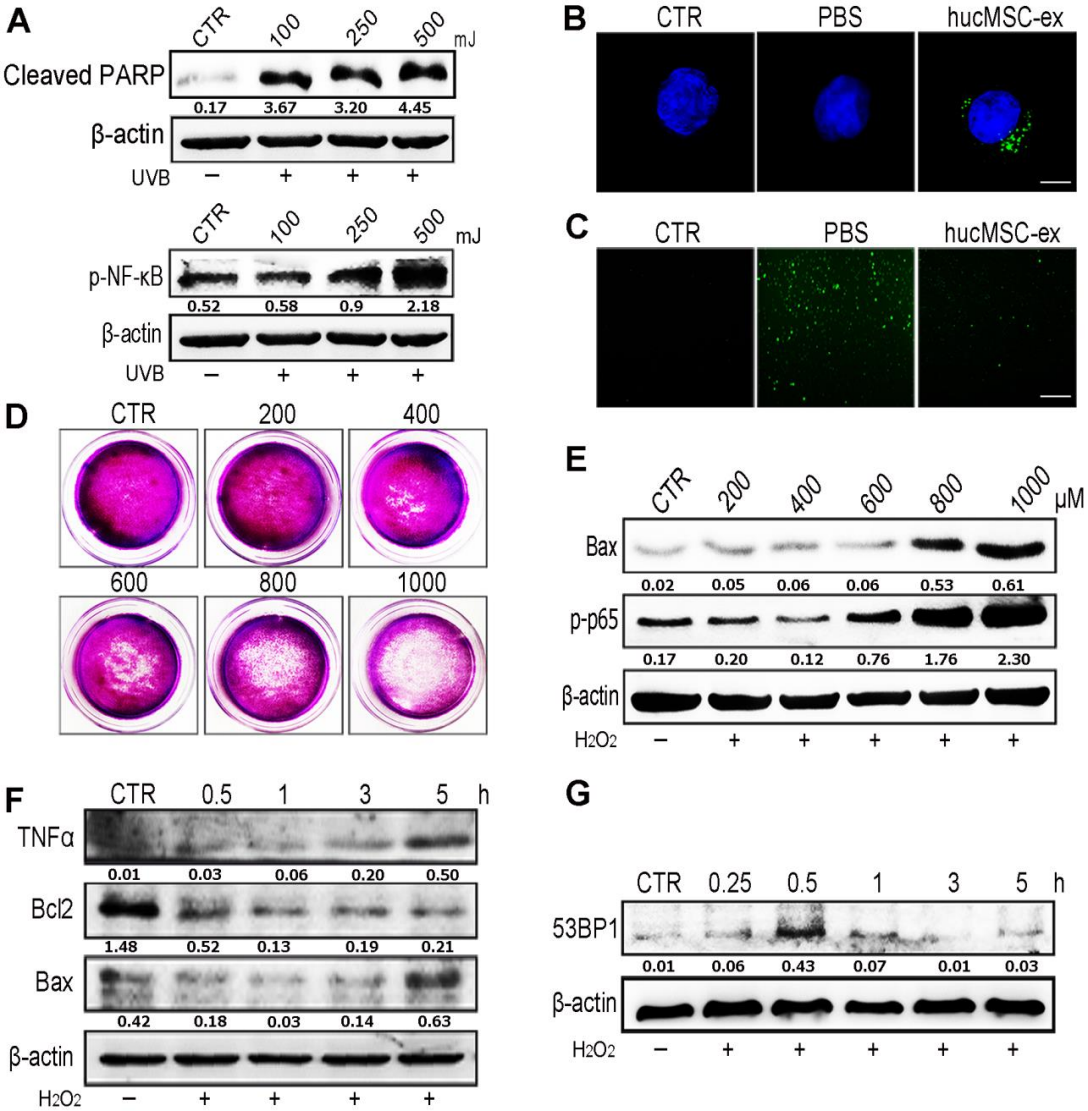
**HucMSC-ex reduced UV-induced ROS production *in vitro*.** (**A**) Western blot was used to detect the expression level of cleaved-PARP and p-NF-κB after different intensity of UV treatment. (**B**) Confocal microscopy observation of the internalization of PKH-67 labeled hucMSC-ex co-incubated with HaCaT for 12 hours. Original magnification (600x). (**C**) ROS production was detected by inverted fluorescence microscope after 500mJ UVB treatment of HaCaT cells (200x). (**D**) Crystal violet detected cell proliferation activity after HaCaT cells treatment with different concentration of H_2_O_2_. (**E**) Western blot analysis of Bax and p-NF-κB expression after HaCaT cells treatment with different concentrations of H_2_O_2._ (**F**) Western blot was used to detect the expression level of inflammatory cytokines and apoptosis in HaCaT cells treated with H_2_O_2_ at different times. (**G**) Western blot detection of 53BP1 expression level of H_2_O_2_ after HaCaT cells treatment at different times.

Oxidative stress has been shown to play an important role in the development of aging and aging-related diseases. UV radiation can produce large quantities of ROS, which are responsible for cellular DNA damage, apoptosis, inflammation, and cellular senescence. Western blot analysis revealed that the application of varying intensities of UV radiation to skin cells caused DNA damage and inflammation in a dose-dependent manner (Figure 3A). We found that DiI-labeled hucMSC-ex could be internalized by HaCaT human keratinocyte cells in a time-dependent manner (Supplementary Figure 4). To examine the dose dependence of these effects, we used various concentrations of hucMSC-ex (200, 400, and 600 μg) to treat a model of acute photodamage induced by UV radiation in the skin (Supplementary Figure 7). Further studies showed that UV exposure resulted in the generation of large quantities of ROS in HaCaT cells; however, hucMSC-ex (600 μg) were internalized by HaCaT cells and significantly downregulated intracellular ROS production (Figure 3B, 3C). As expected, UV radiation-induced ROS play important roles in mediating cell damage and apoptosis, but hucMSC-ex treatment was able to inhibit oxidative stress pathways. Subsequently, we induced oxidative stress in HaCaT cells using different concentrations of H_2_O_2_. After 600 μM H_2_O_2_ treatment, HaCaT cells continued to proliferate and displayed moderate inflammatory responses (Figure 3D, 3E); however, DNA damage and inflammatory responses increased when 600 μM H_2_O_2_ was applied for an increasing length of time (Figure 3F, 3G). We further used the cell-counting kit 8 (CCK8) to detect HaCaT cell proliferation after treatment with different concentrations of H_2_O_2_ (Supplementary Figure 8). With the increase of H_2_O_2_ concentration, the proliferation activity of skin cells decreased gradually. To further explore the role played by hucMSC-ex in the regulation of oxidative stress, we used 600 μM H_2_O_2_ concentrations for all subsequent experiments.

### HucMSC-ex protects skin keratinocytes from oxidative stress

**Figure 4:**
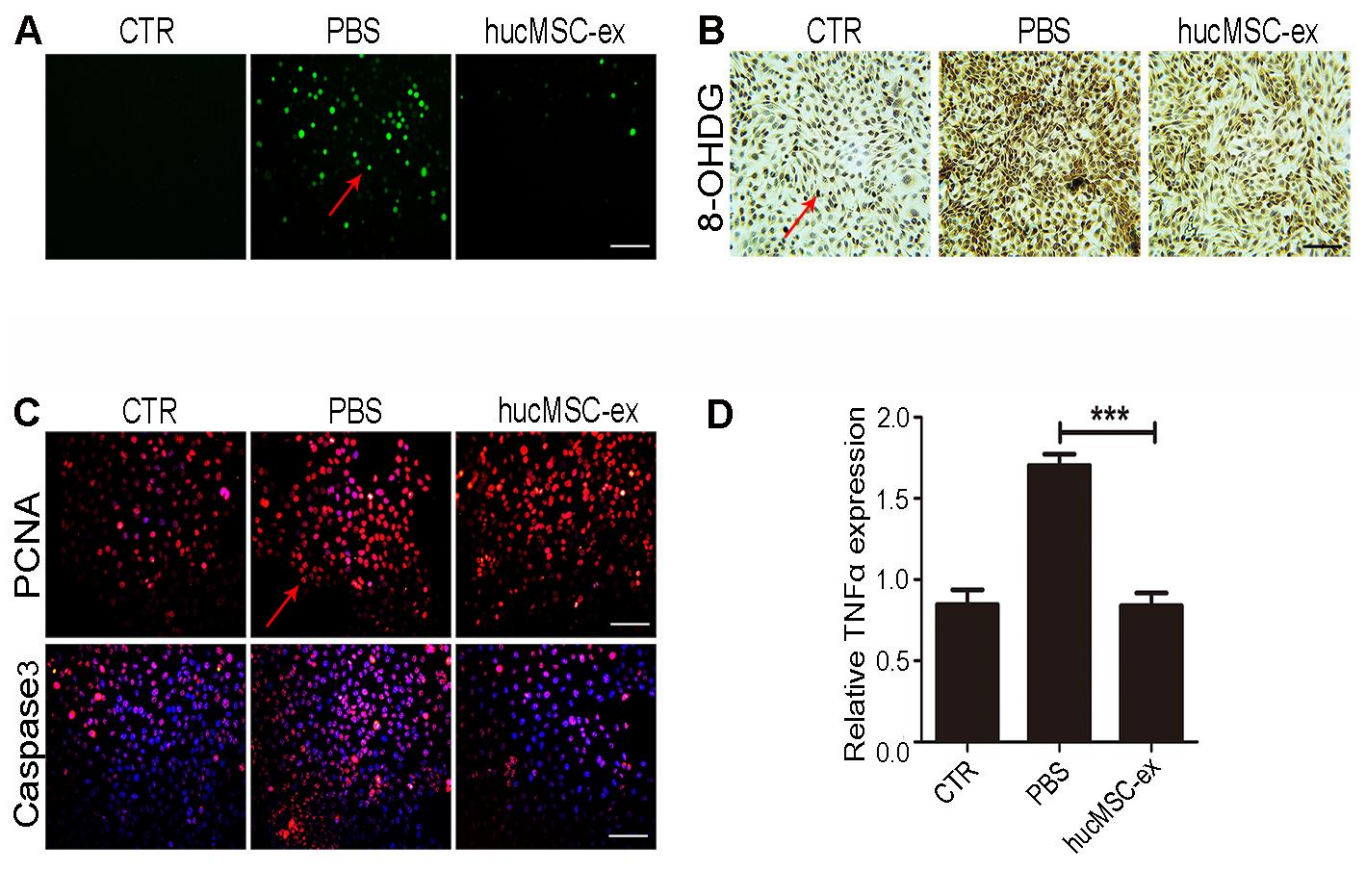
**HucMSC-ex protect skin keratinocytes from oxidative stress.** (**A**) The ROS production of HaCaT cells after 600μM H_2_O_2_ treatment was detected by the inverted fluorescence microscope. (**B**) Immunohistochemical staining of 8-OHDG expression level in HaCaT cells treated with 600μM H_2_O_2_. (**C**) Immunofluorescence detection of PCNA and Caspase3 expression level in HaCaT cells treated with 600μM H_2_O_2_. (**D**) qRT-PCR detection of the expression level of HaCaT cells inflammatory cytokines TNF-α (n = 3; *p < 0.05, **p < 0.01, ***p < 0.001).

Exposure to UV radiation can cause lipid peroxidation, protein modifications, and DNA damage, promoting apoptosis-related signal transduction, contributing to the depletion of skin stem cells and its microenvironment, eventually leading to cell death [19]. We found that hucMSC-ex treatment also significantly reduced H_2_O_2_-induced ROS production compared with that in the PBS group (Figure 4A). The results of immunohistochemical staining showed that the expression levels of 8-OHDG significantly increased after H_2_O_2_ treatment in HaCaT cells, whereas hucMSC-ex treatment significantly reduced the 8-OHDG expression level (Figure 4B). Subsequently, the expression of proliferating cell nuclear antigen (PCNA) and apoptosis-related proteins (Caspase3) was detected by fluorescent immunochemistry. The results showed that 600 μM H_2_O_2_ treatment inhibited HaCaT proliferation and increased apoptosis, whereas hucMSC-ex treatment significantly promoted the proliferation of HaCaT cells and inhibited apoptosis (Figure 4C). Compared with that in the PBS group, quantitative real-time-polymerase chain reaction (qRT-PCR) analysis showed that hucMSC-ex treatment downregulated the expression of the inflammatory cytokine tumor necrosis factor-α (TNF-α) induced by oxidative stress (Figure 4D). These results further demonstrated that hucMSC-ex exerts cytoprotective effects by inhibiting oxidative stress.

### HucMSC-ex treatment promotes SIRT1 expression under oxidative stress conditions and activates autophagy to alleviate HaCaT cell damage

**Figure 5:**
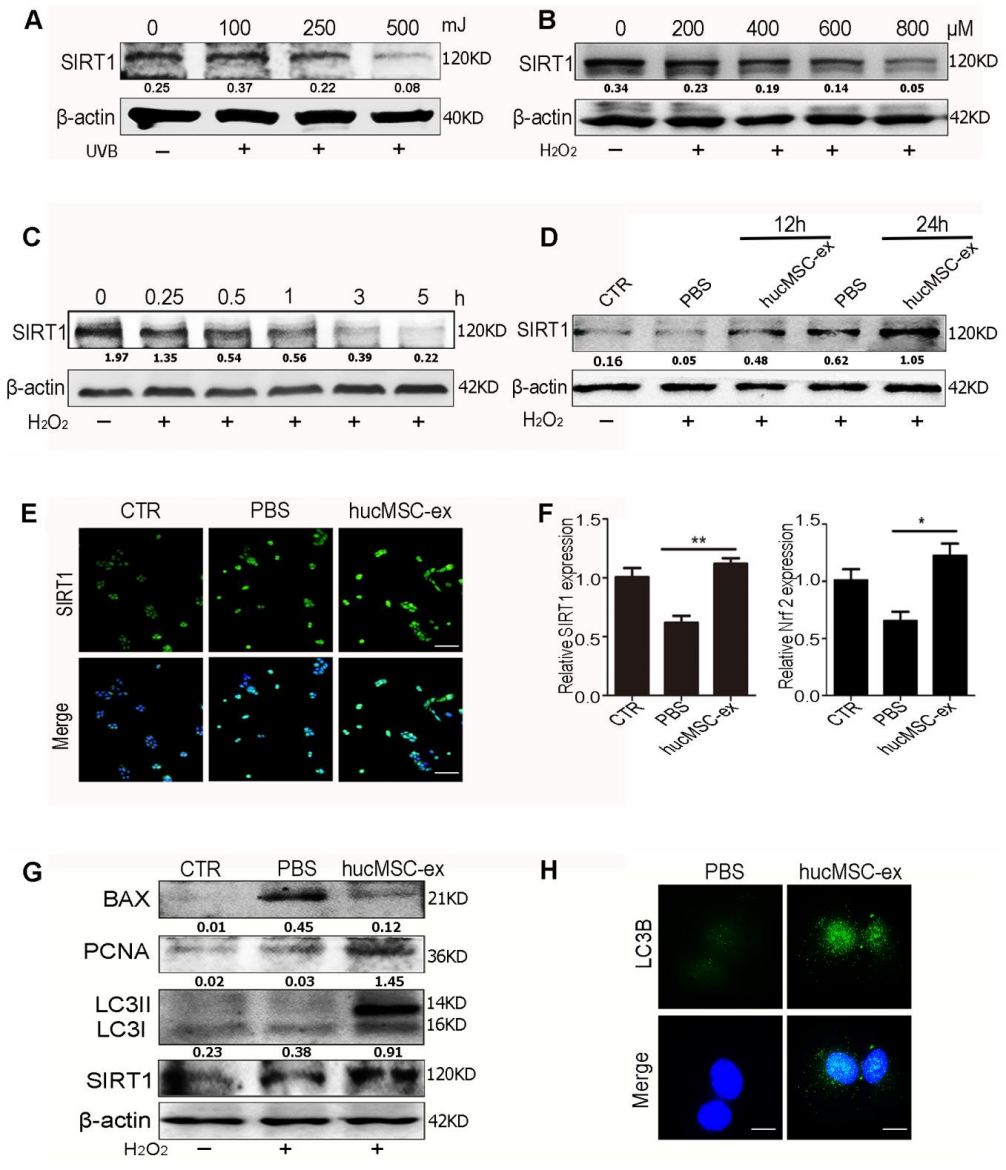
**HucMSC-ex promote SIRT1 expression level under oxidative stress and activates autophagy to alleviate HaCaT cells damage.** (**A**) Western blot analysis of SIRT1 expression level in HaCaT cells treated with different intensity of UVB. (**B**) Western blot analysis of SIRT1 expression level in HaCaT cells treated with different concentrations of H_2_O_2_. (**C**) Western blot detection of SIRT1 expression level in HaCaT cells treated with H_2_O_2_ at different times. (**D**) Western blot detection of SIRT1 expression level after H_2_O_2_ treatment of HaCaT cells at different times. (**E**) Immunofluorescence detection of SIRT1 expression level after H_2_O_2_ treatment of HaCaT cells. (**F**) qRT-PCR was used to detect the expression level of SIRT1 and Nrf2 mRNA of HaCaT cells after 24hours treatment with H_2_O_2_ (n = 3; *p < 0.05, **p < 0.01, ***p < 0.001). (**G**) Western blot detection of PCNA, Bax, LC3II, LC3I and SIRT1 protein expression level. (**H**) Immunofluorescence detection of hucMSC-ex effect on autophagy associated protein LC3B expression level.

We evaluated SIRT1 expression levels under oxidative stress conditions. The results of western blot analysis showed that SIRT1 expression levels were downregulated in a dose-dependent manner after exposure to various doses of UV radiation or H_2_O_2_ (Figure 5A, 5B). Furthermore, SIRT1 expression levels were downregulated in a time-dependent manner after exposure to 600 μM H_2_O_2_ in HaCaT cells (Figure 5C).

These results indicated that SIRT1 expression decreased under oxidative stress conditions. However, hucMSC-ex treatment reversed this phenomenon. The western blot results showed that after 12 and 24 hours of co-treatment with 600 μM H_2_O_2_ and hucMSC-ex, HaCaT cells presented significantly enhanced SIRT1 expression (Figure 5D). The immunofluorescence results also showed that the SIRT1 expression level was more pronounced in the hucMSC-ex group than in the PBS group (Figure 5E). The results of the qRT-PCR analysis confirmed that hucMSC-ex treatment promoted the upregulation of SIRT1 and nuclear factor erythroid 2-related factor 2 (Nrf2) mRNA expression under oxidative stress conditions (Figure 5F). We also detected the activation of autophagy after the co-treatment of HaCaT cells with 600 μM H_2_O_2_ and hucMSC-ex for 24 hours. We found that the ratio of the autophagy-related proteins LC3II/I and PCNA expression were significantly increased, and apoptosis was significantly reduced in the hucMSC-ex co-treatment with H_2_O_2_ treated group (Figure 5G). Immunofluorescence analysis also revealed the increased expression of the autophagy-associated proteins LC3B in the hucMSC-ex treatment group, further indicating that hucMSC-ex may promote autophagy activation by upregulating SIRT1 (Figure 5H). Collectively, our data suggested that hucMSC-ex treatment can protect against oxidative stress-induced DNA damage and prevent apoptosis by upregulating the SIRT1 signaling pathway. These findings confirmed previously reported studies that suggested that SIRT1 promotes cell survival under oxidative state conditions by activating autophagy. Thus, our study further explored the mechanisms through which hucMSC-ex treatment promotes SIRT1 upregulation under oxidative stress conditions.

### HucMSC-ex delivers 14-3-3ζ protein, which promotes SIRT1 expression in HaCaT cells under oxidative stress conditions

**Figure 6:**
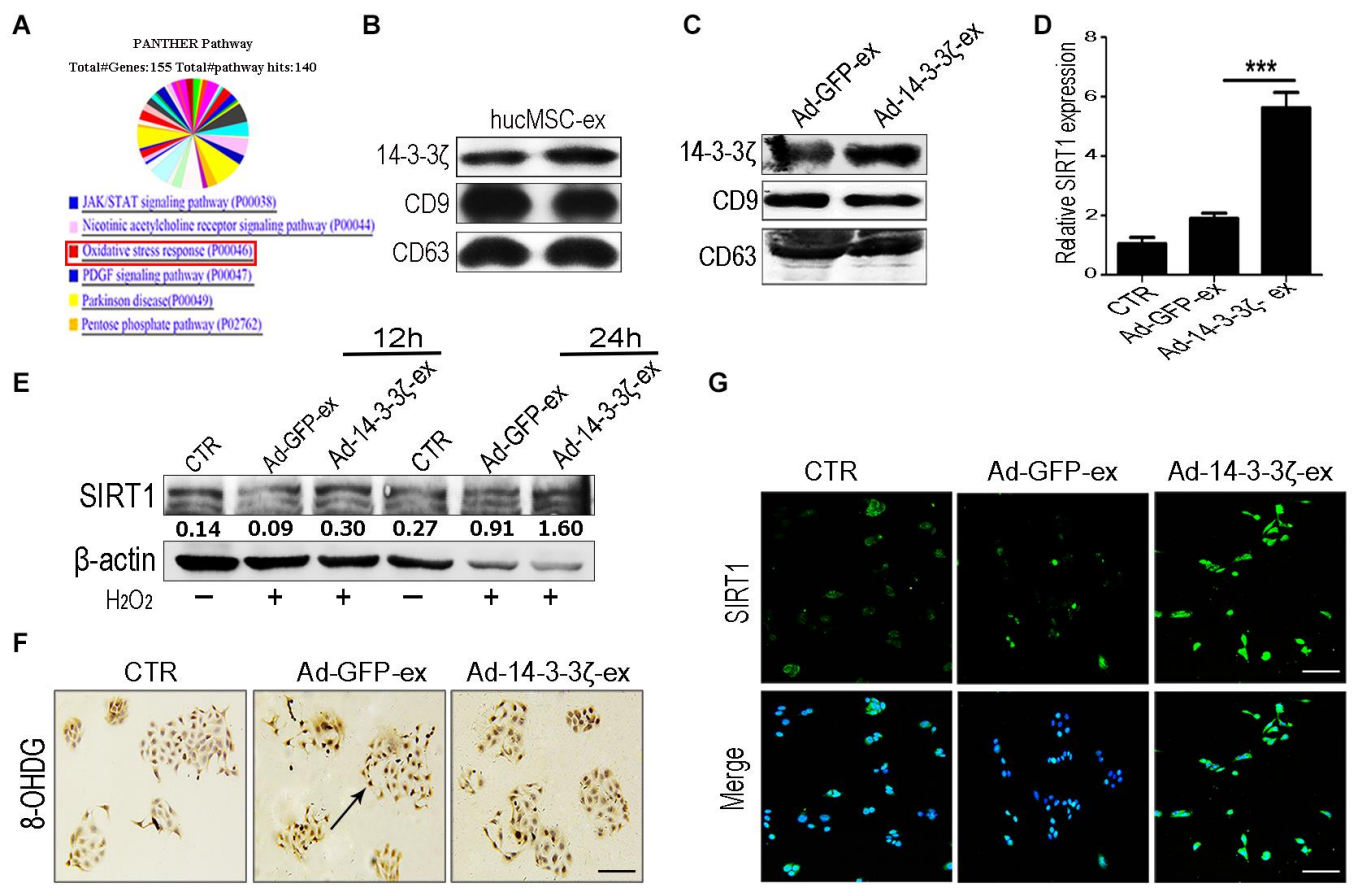
**HucMSC-ex delivered 14-3-3ζ protect HaCaT cells from oxidative stress *in vitro*.** (**A**) Western blot analysis of 14-3-3ζ expression level in Ad-GFP-ex and Ad-14-3-3ζ-ex. (**B**) qRT-PCR quantification of SIRT1 mRNA expression level in HaCaT cells treated with Ad-14-3-3ζ-ex after 24h (n = 3; *p < 0.05, **p < 0.01, ***p < 0.001). (**C**) Immunofluorescence staining detection of SIRT1 expression level in HaCaT cells treated with Ad-14-3-3ζ-ex after 24hours. (**D**) Western blot analysis of SIRT1 expression level in HaCaT cells treated with Ad-14-3-3ζ-ex after 12hours and 24hours. (**E**) The DNA damage of HaCaT cells after treatment with Ad-14-3-3ζ-ex was detected by immunohistochemistry staining. (**F**) Western blot method for the detection of SIRT1 expression level after transfection of HaCaT cells with different titers of adenovirus. (**G**) Immunofluorescence staining detection of SIRT1 expression level after transfection of adenovirus into HaCaT cells.

The 14-3-3 protein can bind SIRT1, which plays an important role in regulating stress and prolonging life [20]. In our previous work, we systematically analyzed the protein composition of hucMSC-ex by using mass spectrometry (Figure 6A and Supplementary Figure 1). Our screening identified that the 14-3-3 family of proteins was abundantly expressed in hucMSC-ex, with the particular enrichment of 14-3-3ζ (Figure 6B). To verify the effects of 14-3-3ζ on SIRT1 protein expression, hucMSCs were transfected with an adenoviral vector carrying the 14-3-3ζ protein expression sequence, and the conditioned medium was collected. The exosomes (Ad-14-3-3ζ-ex) in the supernatant were isolated and purified using previously established methods [13]. The particle sizes and concentrations of exosomes were analyzed by using NTA (Supplementary Figure 2). The western blot analysis showed that the significantly increased expression of 14-3-3ζ protein in Ad-14-3-3ζ-ex, whereas the expression level of 14-3-3ζ protein in hucMSC-ex from hucMSCs transduce with a GFP adenoviral vector (Ad-GFP-ex) showed no change in 14-3-3ζ levels (Figure 6C). To further confirm our hypothesis, HaCaT cells were co-treated with Ad-14-3-3ζ-ex or Ad-GFP-ex and 600 μM H_2_O_2_. The western blot results showed that compared with the PBS group, the co-treatment of HaCaT cells with H_2_O_2_ and Ad-14-3-3ζ-ex for 12 and 24 hours significantly promoted the expression of SIRT1 (Figure 6E). The immunofluorescence and qRT-PCR results further confirmed that Ad-14-3-3ζ-ex treatment significantly promoted the expression of SIRT1 (Figure 6D, 6G). We also designed three small interfering RNAs (siRNAs) targeting SIRT1 to interfere with SIRT1 protein expression in HaCaT cells. The results of the western blotting analysis showed that siRNA1 and siRNA3 were able to effectively silence the expression of SIRT1 protein in HaCaT cells without affecting the expression of 14-3-3ζ protein (Supplementary Figures 5, 6). These results suggested that 14-3-3ζ protein acts as a direct regulatory molecule of SIRT1 protein. We treated SIRT1-knockdown HaCaT cells with exosomes and H_2_O_2_. Compared with the control group, the ROS fluorescence detection results showed no significant change in the expression of ROS in SIRT1-knockdown HaCaT cells treated with hucMSC-ex (Supplementary Figure 6). Immunohistochemistry staining detected DNA damage in HaCaT cells under oxidative stress conditions, and significantly fewer cells presented DNA damage in the Ad-14-3-3ζ-ex treatment group compared with the PBS group (Figure 6F). These results demonstrated that 14-3-3ζ protein, delivered by hucMSC-ex, plays an important role in the regulation of SIRT1.

### Overexpression or knockdown of 14-3-3ζ protein affects SIRT1 expression

**Figure 7:**
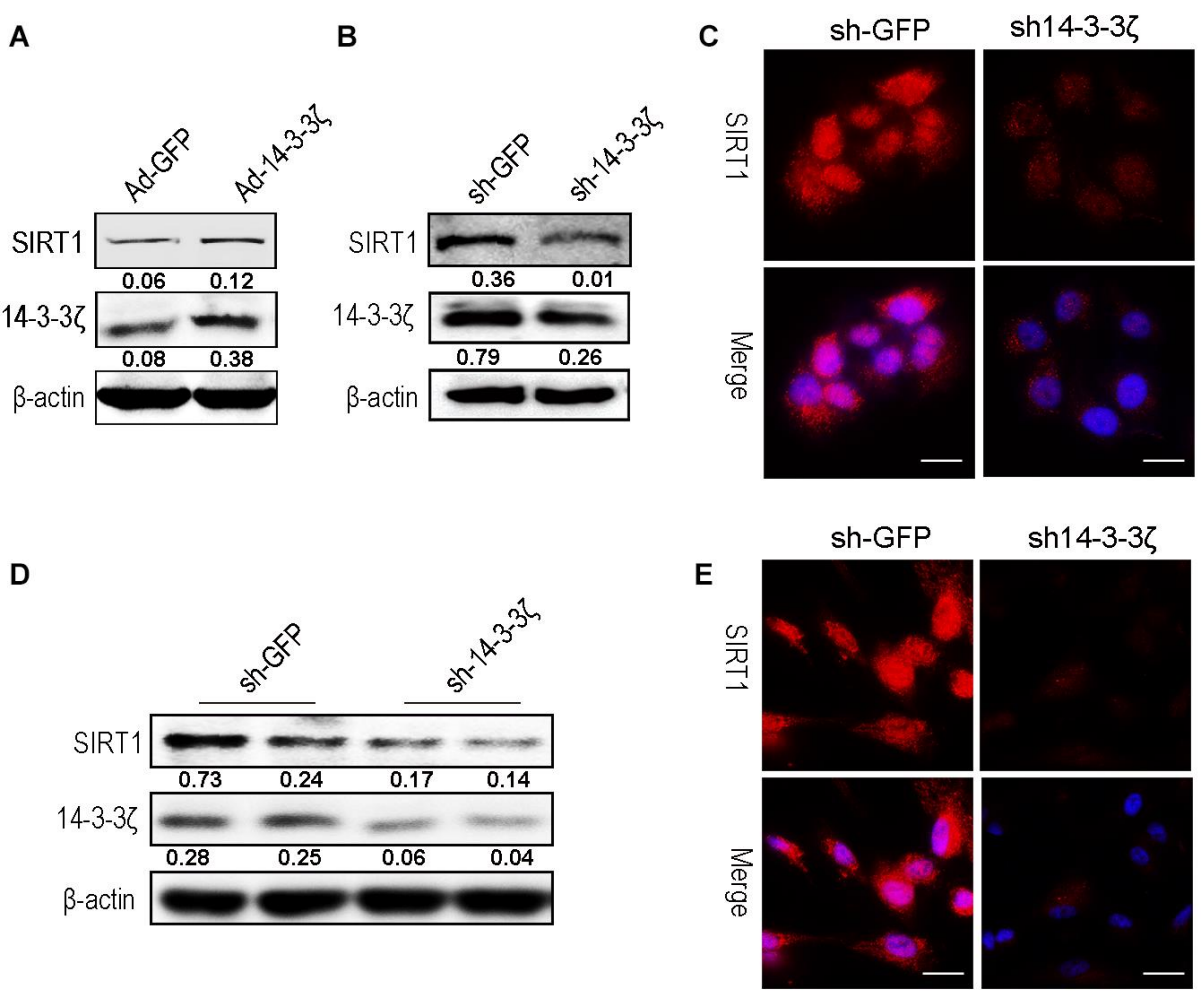
**Knockdown the 14-3-3ζ inhibit SIRT1 expression in HaCaT and hucMSC cells.** (**A**) Western blot analysis of SIRT1 expression level after HaCaT cells were transfected with containing the 14-3-3ζ sequence adenovirus expression vector. (**B**) Western blot analysis of SIRT1 expression level after HaCaT cells were transfected with containing the 14-3-3ζ sequence lentiviral expression vector. (**C**) Immunofluorescence detection of SIRT1 expression level after HaCaT cells were transfected with containing the 14-3-3ζ sequence lentiviral expression vector. (**D**) Western blot analysis of SIRT1 expression level after hucMSCs cells were transfected with containing the 14-3-3ζ sequence lentiviral expression vector. (**E**) Immunofluorescence detection of SIRT1 expression level after HaCaT cells were transfected with containing the 14-3-3ζ sequence lentiviral expression vector.

To further verify that the 14-3-3ζ protein delivered by hucMSC-ex regulates the expression of SIRT1, HaCaT cells were transfected with either an adenovirus to overexpress the 14-3-3ζ protein or a lentiviral vector to knock down the 14-3-3ζ protein. The western blot results revealed that the 14-3-3ζ protein was overexpressed or knocked down in the target cells, respectively, and the expression level of SIRT1 was correspondingly upregulated (Figure 7A) or downregulated (Figure 7B). The immunofluorescence results revealed that the knockdown of the 14-3-3ζ protein resulted in a significant decrease in the SIRT1 protein expression level (Figure 7C). In subsequent experiments, we silenced the 14-3-3ζ protein in hucMSC cells (Supplementary Figure 3), and the western blot and immunofluorescence revealed that the knockdown of the 14-3-3ζ protein resulted in a significant decrease in the SIRT1 protein expression level (Figure 7D, 7E). The above results indicated that the enrichment of 14-3-3ζ protein in hucMSC-ex acts as an important upstream regulatory molecule of the SIRT1 protein.

## DISCUSSION

Both UV radiation and H_2_O_2_ are major factors that can cause skin cell damage [21]. Long-term exposure to excessive UV radiation results in skin photodamage and skin-related diseases. The oxidative free radical theory revealed that UV radiation contributes to skin DNA damage and apoptosis primarily through the generation of large quantities of ROS, which cause the oxidative denaturation of macromolecular substances, such as nucleic acids and proteins [22]. Thus, UV radiation-induced oxidative stress in keratinocytes can trigger many skin diseases, including sunburn, skin cancer, and advanced skin aging. In addition, H_2_O_2_, which is generated in the skin in response to UV radiation, can also induce large quantities of ROS, causing direct oxidative stress in human keratinocytes. Therefore, UV radiation and H_2_O_2_ were used in this study to establish *in vivo* and *in vitro* injury models, respectively.

HucMSCs are an important source of exosomes and have been widely used in the field of regenerative medicine. Numerous basic and clinical studies have indicated that hucMSC-ex can exert anti-inflammatory, anti-oxidation, and anti-apoptosis effects, promoting proliferation [23]. In this study, we investigated the role played by hucMSC-ex treatment in UV radiation-induced skin photodamage. We found that three consecutive days of UV radiation induced skin tissue redness, scaling, and inflammatory cell infiltration. The subcutaneous injection of hucMSC-ex protected skin cells against UV radiation-induced DNA damage, inflammation, and apoptosis. However, the mechanisms through which hucMSC-ex exerts these effects requires further investigation.

Hydrogen peroxide, hydroxyl radicals, and superoxide anions induce oxidative stress, causing DNA damage and cell death. The SIRT1/Nrf2 signaling pathway plays an important role in the inhibition of inflammation, the reduction of oxidative stress, and the delay of aging [24–27]. SIRT1 is a member of a highly conserved gene family encoding NAD^+^-dependent deacetylases and is widely expressed in mature tissues throughout the body [28], especially in early embryonic and germ cells. SIRT1 has been demonstrated to participate in various cellular pathways, including cell metabolism [29], cellular senescence [30], endocrine signaling [31], stress responses [32], and the regulation of cell death and survival [33]. SIRT1 deacetylates not only histones but also many nonhistone proteins involved in transcriptional activities, such as p53 [18], Ku70 [34], Forkhead box O (FOXO) [35], and peroxisome proliferator-activated receptor γ (PPARγ) [36]; thus, SIRT1 plays an important role in the repression of apoptosis and increased cell survival. SIRT1 activation has been reported to accelerate the clearance of ROS, promote the activation of autophagy, enhance the DNA repair ability of cells, and protect cells from apoptosis [37].

**Figure 8:**
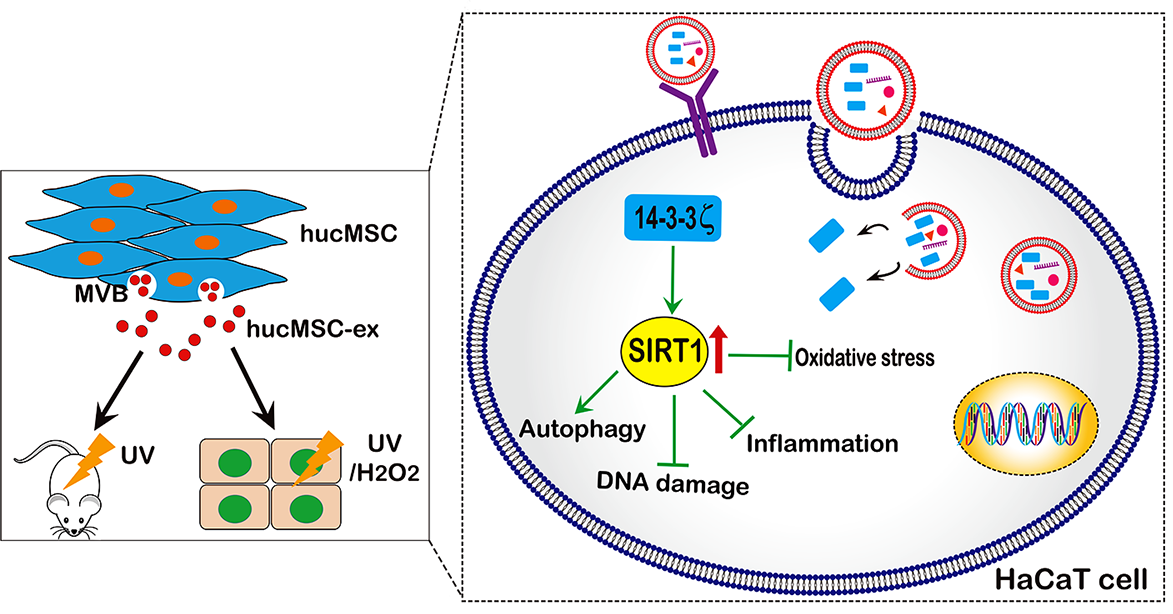
**A proposed model for hucMSC-ex-specific 14-3-3ζ confer protection against UV-induced acute photodamage.** hucMSC-ex play an important role of cytoprotection through inhibit oxidative stress and inflammatory, reduce UV-induced DNA damage, and promote autophagy activation *in vivo* and *in vitro*. Mechanically, the 14-3-3ζ proteins were carried by hucMSC-ex are responsible for up-regulation of SIRT1 expression.

Our research showed that UV radiation and H_2_O_2_ could downregulate SIRT1 in a time- and dose-dependent manner. However, hucMSC-ex treatment reversed this phenomenon. We also found that the SIRT1 protein level, the ratio of the autophagy-related proteins LC3II/I, and the PCNA expression level were significantly increased, and apoptosis was significantly reduced in the hucMSC-ex treated group compared with the control group. This result further indicated that hucMSC-ex treatment could upregulate SIRT1 expression level to promote autophagy activation. Previously, SIR-2.1 was reported to bind to DAF-16 in a 14-3-3-dependent manner. In the absence of 14-3-3 protein, SIR-2.1 was unable to bind DAF-16; thus, DAF-16 remained inactive, despite a nuclear localization, resulting in a shorter life span and enhanced sensitivity to stress [38]. In addition, several studies have shown that the 14-3-3 family proteins protect against stress-induced apoptosis [39–41]. Our previous study confirmed that 14-3-3 family proteins (including14-3-3ζ, 14-3-3θ, and 14-3-3η) are abundantly expressed in hucMSC-ex, with the particular enrichment of 14-3-3ζ proteins [16]. 14-3-3τ binds to the phosphorylation site Ser31 of the transcription factor E2F1 during DNA damage, inhibiting the ubiquitination-mediated degradation of E2F1 and inhibiting cell apoptosis [42]. E2F1 is an important and positive regulator of the cell cycle, promoting the progression of cells from the G_0_/G_1_ phase to the S phase [43]. Studies have confirmed the existence of two E2F1 binding sites in the promoter region of the SIRT1 gene; therefore, E2F1 is thought to significantly promote the transcriptional expression of SIRT1 [44]. Thus, we speculate that 14-3-3ζ protein can bind and stabilize E2F1 to further promote SIRT1 expression, playing an important role in the regulation of stress and the prolongation of life. Our data indicated that abundant 14-3-3ζ protein was found in hucMSC-ex, and 14-3-3ζ protein overexpression in hucMSC-ex promoted SIRT1 expression and enhanced the antioxidant protective effects of hucMSC-ex *in vitro*; therefore, hucMSC exosomal 14-3-3ζ protein may be critical for the hucMSC-ex antioxidant-induced recovery from skin injury. In addition, the overexpression of 14-3-3ζ protein in HaCaT cells also resulted in increased SIRT1 expression levels. In brief, the presented data further confirmed that hucMSC-ex could provide antioxidant cytoprotective effects against UV radiation and H_2_O_2,_ both *in vitro* and *in vivo*, likely mediated by the delivery of 14-3-3ζ protein, which reduces ROS production and inhibits oxidative stress-induced apoptosis via the upregulation of the SIRT1 pathway (Figure 8). Thus, our study also suggested that hucMSC-ex may represent a novel therapeutic strategy for preventing oxidative damage and a therapeutic agent in skin aging.

## CONCLUSIONS

In summary, our results demonstrated that the topical application of hucMSC-ex could reduce UV radiation-induced ROS production and DNA damage and promote the activation of autophagy to exert cytoprotective effects. These findings showed that hucMSC-ex could upregulate SIRT1 expression levels in HaCaT cells by trafficking 14-3-3ζ protein to relieve UV radiation- and H_2_O_2_-induced oxidative stress damage. Therefore, hucMSC-ex could be exploited as a new therapeutic tool for the prevention and treatment of skin photodamage.

## MATERIALS AND METHODS

### Ethics

All the experimental protocols were approved by the Medical Ethics Committee of Jiangsu University (2012258).

### Cell culture

HucMSCs were isolated and characterized, as previously described [45]. Briefly, fresh human umbilical cord tissues were collected from the affiliated hospital of Jiangsu University and processed into 1 mm^3^ tissue blocks within 6 hours of collection. HucMSCs were cultured in minimal essential medium alpha (MEM-α) containing 10% fetal bovine serum (FBS, Gibco, Grand Island, USA) at 37° C with 5% CO_2_. The cells in this experiment were from passages 3–4. Skin keratinocyte HaCaT cells were purchased from American Type Culture Collection (ATCC) and maintained in high-glucose Dulbecco’s modified Eagle`s medium (DMEM) with 10% fetal bovine serum at 37° C with 5% CO_2_.

### Osteogenic and adipogenic differentiation *in vitro*

HucMSCs in passage 3 were seeded in 6-well plates containing either osteogenic (0.1 mM dexamethasone, 10 mM β-glycerophosphate, and 50 mM ascorbate-phosphate) or adipogenic medium (Cyagen Biosciences, CA, USA) for 2 weeks, according to the manufacturer’s instructions. After two weeks, the osteogenic and adipogenic differentiation potentials were assessed through Alizarin Red and Oil Red O staining, respectively.

### Purification and characterization of hucMSC-ex

Exosomes were isolated and purified by differential ultracentrifugation, as previously described [46]. The final exosome pellet was resuspended in PBS and subsequently passed through a 0.22-μm filter. The pellet was stored at –80° C for future use. The protein contents of the purified exosomes were detected by bicinchoninic acid (BCA) protein assay kit (CWBIO). The final concentration of hucMSC-ex for *in vitro* use was 600 μg, whereas 1 mg was used for *in vivo* studies. The morphologies of exosomes were observed by TEM (FEI Tecnai 12, Philips, Netherlands). The sizes and concentrations of exosomes were detected by using NTA (NanoSight, Amesbury, UK). The expression of exosomal surface markers, including CD9, CD81, and TSG101, and the lack of calnexin expression were determined by western blot analysis.

### Establishment of rat acute skin photodamage model

Adult female Sprague Dawley (SD) rats (weighing 180 ± 20 g) were purchased from the Animal Centre of the Chinese Academy of Sciences (Shanghai, China). The rats were maintained under suitable environmental conditions (temperature 25° C, humidity 50%, 12 hour:12 hour light:dark cycle) with sufficient access to food and water. The rats were anesthetized with chloral hydrate at a dose of 40 mg/kg. A 3 cm^2^ region of back hair was removed, and the skin was exposed to UV radiation at 3 times the minimum erythema dose.

### HucMSC-ex labeling and internalization

HucMSC-ex were labeled with the membrane dye PKH-67 (Green), according to the manufacturer’s protocol. Exosomes in suspension were mixed with PKH-67 in the dark at 37° C for 30 min. The labeled exosomes were washed with PBS and filtered through a 100-kDa-molecular-weight cut-off ultrafiltration membrane (Millipore) at 1000 × g for 30 min to remove the unbound dye. PBS was used as a negative control. HaCaT cells (1 × 10^4^ per well) were seeded in 12-well plates and incubated with PKH-67-labeled exosomes at 37° C for 12 hours. The cells were washed with 4° C pre-cooled PBS and fixed in 4% paraformaldehyde. Nuclei were counterstained with Hoechst 33342 (1:300). A confocal microscope was used to acquire sequentially fluorescent images (Thermo Fisher Scientific).

### qRT-PCR

Total RNA from HaCaT cells was extracted with TRIzol reagent. cDNA was reversed transcribed, according to the SuperScript^TM^ II RT kit manufacturer’s instructions (Invitrogen). The qRT-PCR was used to detect the expression levels of the target genes. β-actin was used as the endogenous control. The specific primers were produced by Invitrogen (Shanghai, China), and their products are shown in Supplementary Table 1. Three siRNA sequences targeting the SIRT1 gene (Genepharma, Suzhou) were also summarized in Supplementary Table 1.

### UV radiation- and H_2_O_2_-induced HaCaT cell oxidative stress injury model *in vitro*

HaCaT cells were seeded in a six-well plate (1 × 10^5^ cells per well) until they reached 70% confluence. Before UV radiation, the cells were washed with 1 ml PBS and suspended in 0.5 ml PBS. The cells were subsequently irradiated at 500 mJ/cm^2^ intensity, without the plastic dish lid. After UV irradiation, the cells were treated with basal medium containing 600 μg exosomes for 12 or 24 hours. A separate group of HaCaT cells was treated with basal medium containing 600 μM H_2_O_2_ and exosomes for 12 or 24 hours. PBS was used as a negative control. After exosome treatment, the cells were collected, and total RNA and proteins were detected, or the samples were fixed in 4% paraformaldehyde solution for histology analysis.

### ROS measurement

The production of cellular ROS was measured with a nonfluorescent, cell-permeating compound, 2′-7′-dichlorofluorescein diacetate (DCF-DA) (Beyotime). DCF-DA is hydrolyzed by intracellular esterases and oxidized by ROS into a fluorescent compound, DCF. To measure ROS in HaCaT cells, the cells were collected and incubated with DCF-DA (10 mM) for 30 min at 37° C. After washing with PBS, intracellular fluorescence was observed with an Olympus Fluorescent Microscope (200×).

### Western blot

Total proteins from tissues and cells were harvested, washed, and lysed in radioimmunoprecipitation buffer (RIPA) buffer. The protein concentration of each purified exosome sample was determined using a bicinchoninic acid (BCA) protein assay kit (CWBIO). Equal amounts of tissue or cell lysates were separated by 12% sodium dodecyl sulfate-polyacrylamide gel electrophoresis (SDS-PAGE) and transferred onto polyvinylidene fluoride (PVDF) membranes. After being blocked with 5% skim milk for 1 h, the membranes were incubated with primary antibodies and horseradish peroxidase (HRP)-conjugated secondary antibodies and detected using an enhanced chemiluminescent (ECL) substrate detection system. The primary antibodies used in the experiments were as follows: CD9 (1:500, Bioworld Technology, USA), CD63 (1:500, Bioworld Technology, USA), Bax (1:500, Bioworld Technology, USA), Bcl2 (1:500, Bioworld Technology, USA), SIRT1 (1:500, Bioworld Technology, USA), PCNA (1:500, CST, USA), 14-3-3ζ (1:500, Bioworld Technology, USA), TNF-α (1:500, Bioworld Technology, USA), Cleaved caspase3 (1:400, Bioworld, USA) GAPDH (1:2000, CWBIO, China), β-actin (1:2000, CWBIO, China). Primary antibodies were incubated overnight at 4° C. The HRP-conjugated goat anti-rabbit and goat anti-mouse secondary antibodies (1:2000, CWBIO, China) were incubated 2 hours at 37° C.

### Immunohistochemistry and immunocytochemistry staining

Skin tissues were fixed in 4% formaldehyde solution (pH 7.4) and processed into 4-μm-thick paraffin sections. Immunohistochemical staining was performed according to the manufacturer’s protocols (Boster, Wuhan, China). The prepared skin slides were incubated with 8-OHDG primary antibodies (1:50, Japan Institute for Control of Aging) at 4° C overnight. The HaCaT cells were fixed in 4% paraformaldehyde solution (pH 7.4) for 30 min, permeabilized for 15 min with 0.1% Triton-X100, blocked for 30 min with 5% bovine serum albumin, and incubated with 8-OHDG primary antibodies at 4° C overnight. The tissue sections or cell slides were washed and incubated with secondary antibodies. The tissue sections were visualized with 3, 3'-diaminobenzidine and counterstained with hematoxylin. The images of tissue and cell sections were acquired by high-power light microscopy (Nikon, Tokyo, Japan).

### Immunofluorescence staining

HaCaT cells were seeded in a six-well plate, grown to reach 50% confluence, and then co-cultured for 24 hours with 600 μM H_2_O_2_ and 600 μg hucMSC-ex. Cells were fixed in 4% paraformaldehyde for 30 min, permeabilized for 15 min with 0.1% Triton-X100, blocked for 30 min with 5% bovine serum albumin, and further incubated with rabbit monoclonal anti-SIRT1 (1:50, Bioworld Technology, USA), 14-3-3ζ (1:50, Bioworld Technology, USA) and LC3B (1:50, Bioworld Technology, USA) overnight at 4° C, followed by incubation with fluorescein isothiocyanate (FITC)-labeled anti-rabbit IgG secondary antibody (1:200) at 37° C for 30 min. The nuclei were counterstained with Hoechst 33342 (1:300; Sigma-Aldrich). The sections were observed with a fluorescent microscope (Nikon, Tokyo, Japan).

### H&E staining

To detect the extent of skin injury, skin tissues were fixed in 4% paraformaldehyde (pH 7.4), gradually dehydrated, embedded in paraffin, cut into 4-μm sections, and subjected to hematoxylin and eosin (H&E) staining.

### Overexpression or knockdown of 14-3-3ζ in hucMSCs

An adenovirus expression vector containing the 14-3-3ζ (Ad-14-3-3ζ) expression sequence (Geneway, Shanghai, China) was transfected into hucMSCs, according to manufacturer’s guidelines, and adenovirus empty vector (Ad-GFP) was used as the negative control. After 24 hours of transfection, the normal complete medium was replaced. Exosomes were isolated from Ad-14-3-3ζ hucMSCs conditioned medium as previously described.

A lentiviral expression vector containing the 14-3-3ζ small hairpin RNA (shRNA) sequence (GeneChem, Shanghai, China) was selected to target the 14-3-3ζ genes for silencing (Lenti-14-3-3ζ shRNA). Lenti-GFP shRNA was used as the negative control vector. The Lenti-14-3-3ζ shRNA vectors were generated by ligating the vector Tet-pLKO-puro with 14-3-3ζ shRNA oligonucleotides. The shRNA 14-3-3ζ oligonucleotide sequences were as follows: forward, 5’-CCGGGCAGAGAGCAAAGTCTTCTATCTCGAGATAGAAGACTTTGCTCTCTGCTTTTTG-3’ and reverse, 5’-AATTCAAAAAGCAGAGAGCAAAGTCTTCTATCTCGAGATAGAAGACTTTGCTCTCTGC-3’. HucMSCs were transduced with the prepared lentivirus (Lenti-14-3-3ζ shRNA or Lenti-GFP shRNA). Stable cell lines were obtained after selection with 1 μg/ml puromycin (Invitrogen) for 15 days. shRNA expression was induced by adding 80 μg/ml doxycycline. The efficiency of 14-3-3ζ knockdown was evaluated through western blot.

### Statistical analysis

All data are presented as the mean ± standard deviation (SD). Significant differences between groups were analyzed using analysis of variance or Student’s t-test using Prism software (GraphPad, San Diego, USA). *P-value* < 0.05 level was defined as significant.

## Supplementary Material

Supplementary Figures

Supplementary Table 1
