# Supplementary material for: HucMSC exosome-delivered 14-3-3ζ alleviates ultraviolet radiation-induced photodamage via SIRT1 pathway modulation
Source: Aging (Albany NY). 2021 Apr 21;13(8):11542–63. doi: 10.18632/aging.202851 (PMC8109102; doi:10.18632/aging.202851)
Supplement: Supplementary Figures [file aging-13-202851-s001.pdf]

SUPPLEMENTARY FIGURES

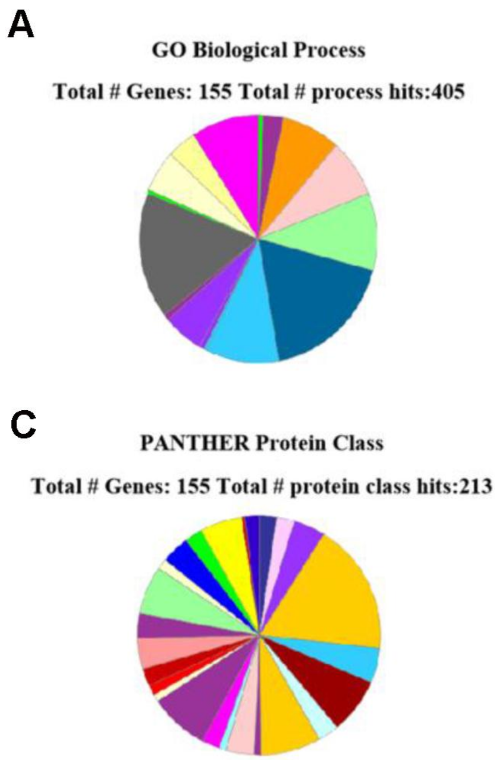

Supplementary Figure 1. The results of detection and analysis of protein composition and function in hucMSC-ex were obtained by LC-MS/MS. (**A**) The biological process of protein mass spectrometry detection results of hucMSC-ex was analyzed by GO enrichment analysis. (**B**) The molecular function of protein mass spectrometry detection results of hucMSC-ex was analyzed by GO enrichment analysis. (**C**) The PANTHER protein class of protein mass spectrometry detection results of hucMSC-ex was analyzed by GO enrichment analysis." to the manuscript.

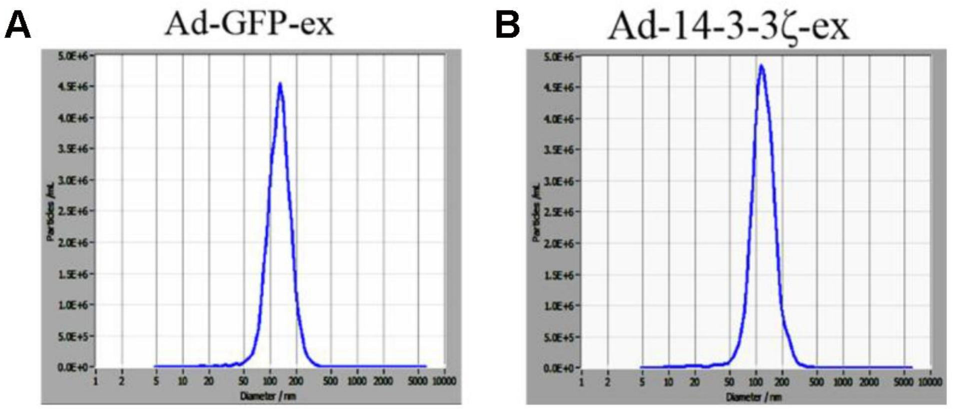

Supplementary Figure 2. The hucMSC-ex size distribution was analyzed using nanoparticle tracking analysis (NTA) with ZetaView_Particle Metrix. (**A**) NTA was used to detect the size distribution of hucMSC-ex overexpressing the empty adenovirus vector. (**B**) NTA was used to detect the size distribution of hucMSC-ex overexpressing the 14-3-3ζ adenovirus vector.

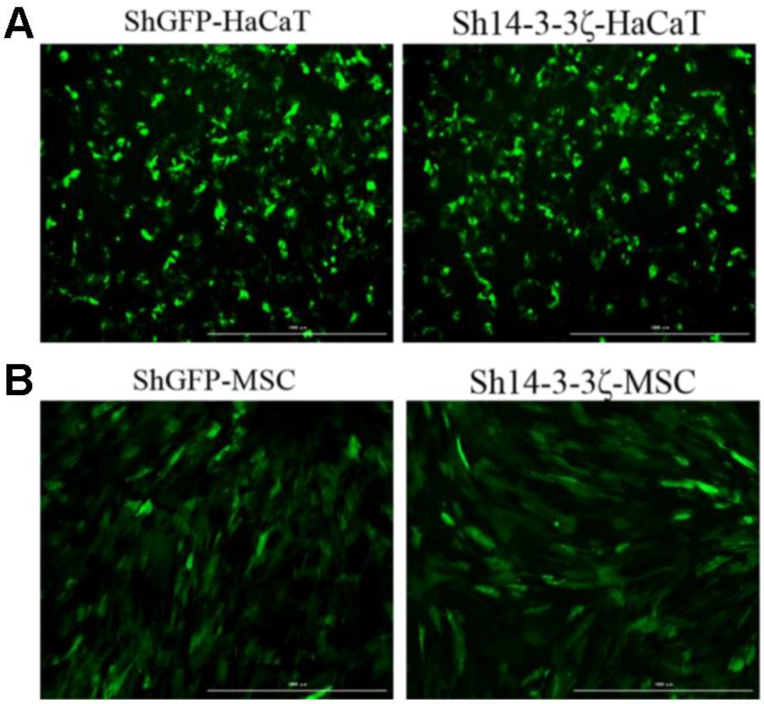

Supplementary Figure 3. Immunofluorescence microscopy was used to detect the expression efficiency of lentivirus transduction HaCaT and hucMSC cells. (**A**) Automatic microplate reader Cytation 5 was used to detect the transfection efficiency of empty vector and knockdown 14-3-3ζ lentivirus into HaCaT cells. (**B**) Automatic microplate reader Cytation 5 was used to detect the transfection efficiency of empty vector and knockdown 14-3-3ζ lentivirus into hucMSC cells.

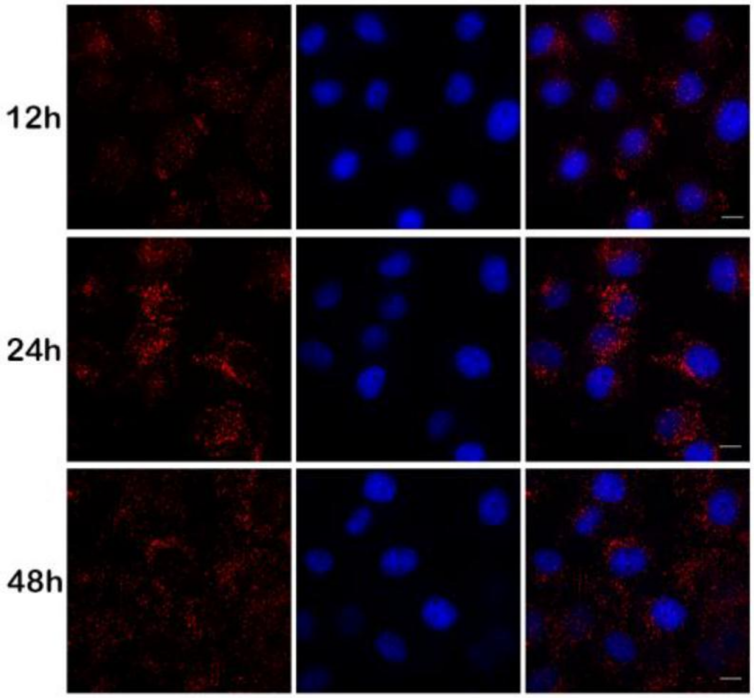

Supplementary Figure 4. Confocal microscopy was used to detect the uptake of DIL-labeled exosomes into HaCaT cells at 12h, 24h, and 48h after UV radiation.

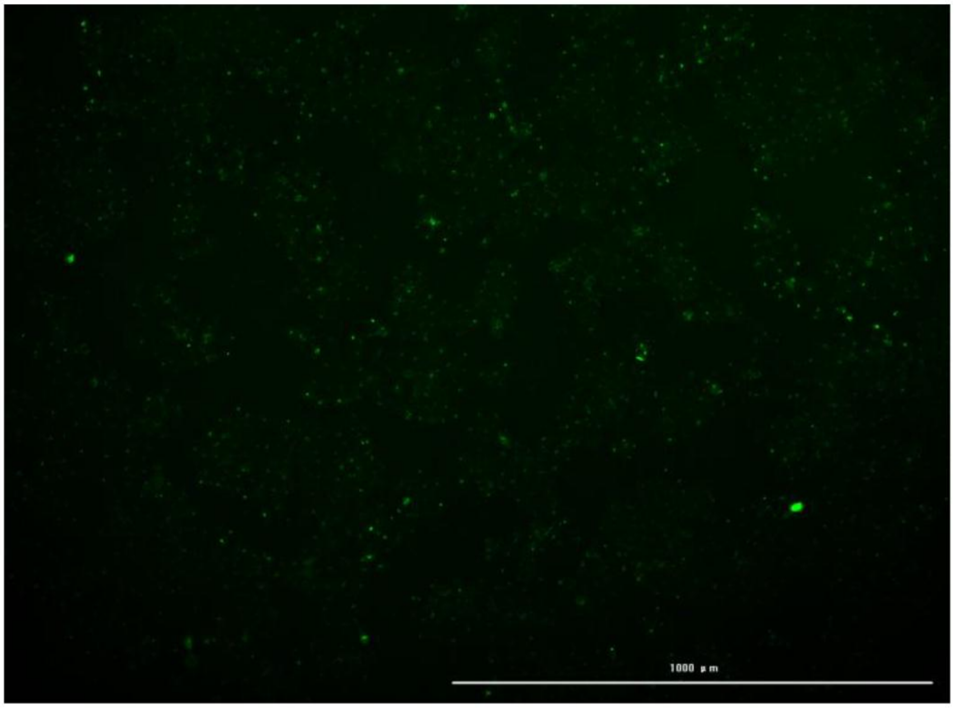

Supplementary Figure 5. After transfection of HaCaT cells with FAM-labeled siRNA for 48h, the transfection efficiency was determined by Automatic microplate reader Cytation 5.

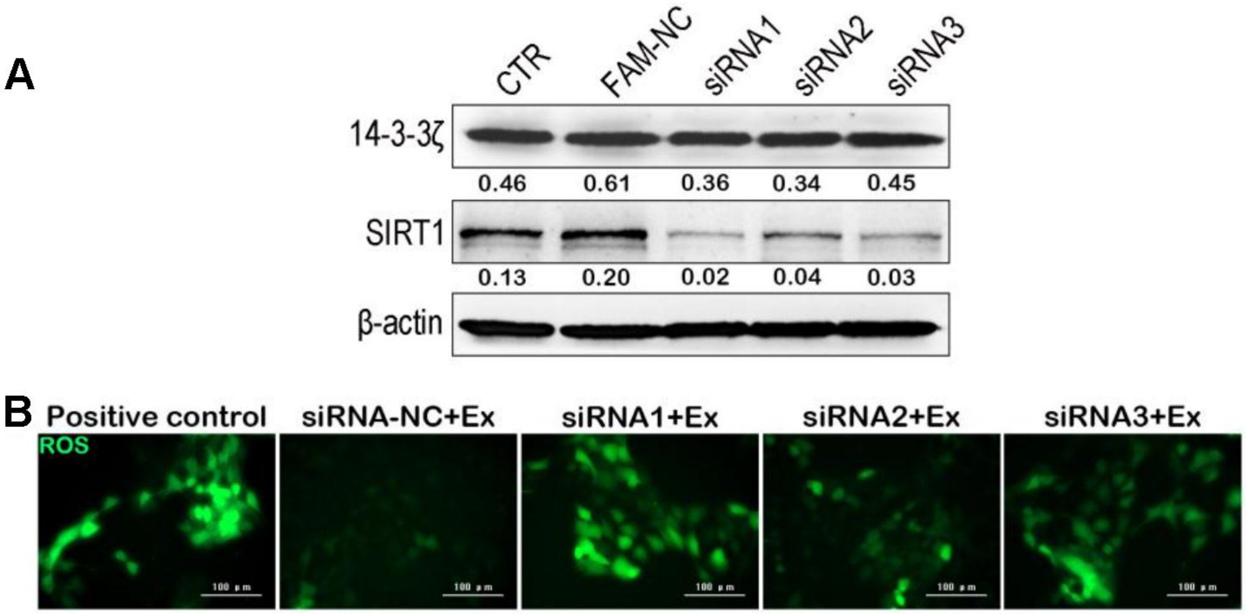

Supplementary Figure 6. After the small interfering RNA knocked down SIRT1 in HaCaT cells, the ability of hucMSC-ex to inhibit ROS production was partially inhibited. (**A**) Western blot was used to detect 14-3-3ζ protein changes in HaCaT cells after transfection of SIRT1 siRNA. (**B**) Automatic microplate reader Cytation 5 was used to detect the levels of ROS in SIRT1 siRNA knockdown HaCaT cells treated with hucMSC-ex and hydrogen peroxide.

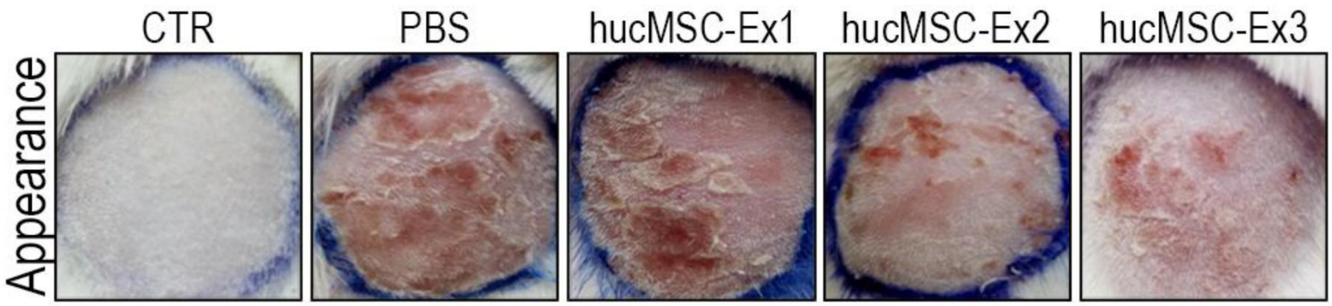

Supplementary Figure 7. HucMSC-ex were dose-dependent for skin photodamage repair in an animal model of uV-induced skin photodamage.

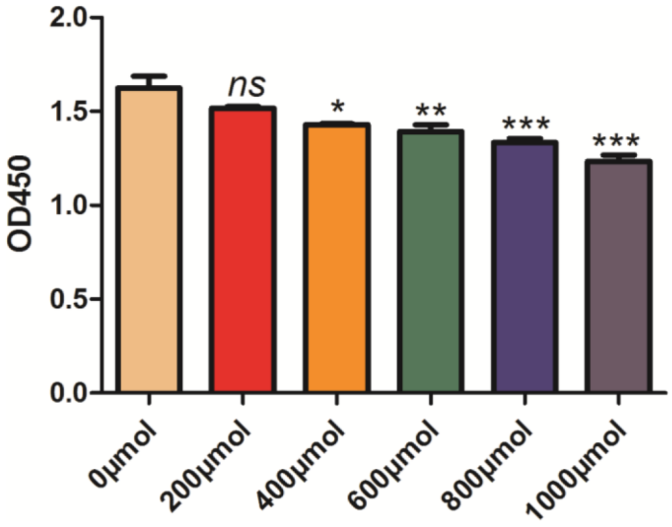

Supplementary Figure 8. CCK8 was used to detect the cell viability of HaCaT cells treated with different concentrations of hydrogen peroxide.
