# Supplementary material for: HucMSC exosome-delivered 14-3-3ζ alleviates ultraviolet radiation-induced photodamage via SIRT1 pathway modulation
Source: Aging (Albany NY). 2021 Apr 21;13(8):11542–63. doi: 10.18632/aging.202851 (PMC8109102; doi:10.18632/aging.202851)
Supplement: Supplementary Table 1 [file aging-13-202851-s002.pdf]

SUPPLEMENTARY TABLE

Supplementary Table 1. Real-time quantitative PCR primer sequence and SIRT1 small interfering RNA sequence.

Gene (human)	Primer	Sequences (5′-3′)	Annealing temperature	Fragment size (bp)
SIRT1	Forward primer	TGCTGGCCTAATAGAGTGGCA	60° C	102
	Reverse primer	CTCAGCGCCATGGAAAATGT		
TNF-α	Forward primer	CCGAGTGACAAGCCTGTAGC	60° C	260
	Reverse primer	AGGAGGTTGACCTTGGTCTG		
Nrf2	Forward primer	TTCCGTCGCTGACTGAAG	60° C	129
	Reverse primer	CGGTCCACAGCTCATCAT		
β-actin	Forward primer	GACCTGTACGCCAACACAGT	60° C	129
	Reverse primer	CTCAGGAGGAGCAATGATCT		
siRNA-SIRT1-homo-512	sense	CCAUCUCUCUGUCACAAAUTT		
	antisense	AUUUGUGACAGAGAGAUGGTT		
siRNA-SIRT1-homo-606	sense	CGGGAAUCCAAAGGAUAAUTT		
	antisense	AUUAUCCUUUGGAUUCCCGTT		
siRNA-SIRT1-homo-1216	sense	CCAAGCAGCUAAGAGUAAUTT		
	antisense	AUUACUCUUAGCUGCUUGGTT		
